# *QuickStats:* Age-Adjusted Drug Overdose Death Rates* Involving Cocaine,^†^ by Region^§^ — National Vital Statistics System, United States, 2021

**DOI:** 10.15585/mmwr.mm7241a4

**Published:** 2023-10-13

**Figure:**
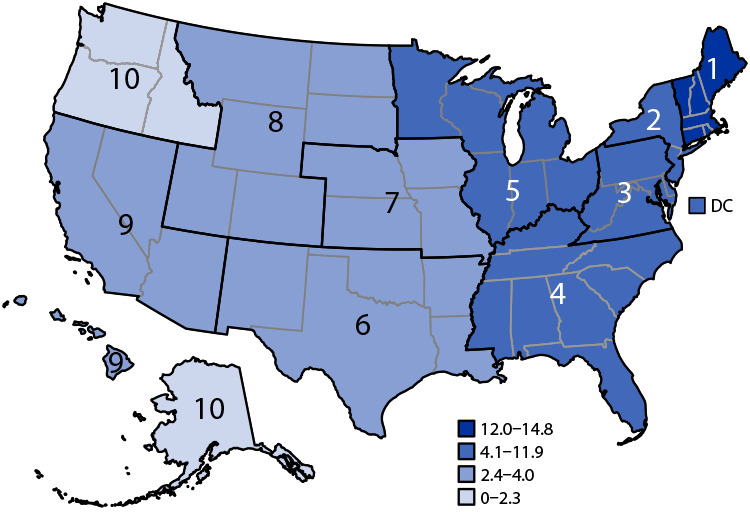
In 2021, the U.S. age-adjusted drug overdose death rate involving cocaine was 7.3 deaths per 100,000 standard population. Rates were higher in HHS regions 1–5 (mostly areas east of the Mississippi River) and were lower in regions 6–10 (areas west of the Mississippi River). The highest rate was in Region 1 (14.8), which includes Connecticut, Maine, Massachusetts, New Hampshire, Rhode Island, and Vermont. The lowest rate was in Region 10 (2.3), which includes Alaska, Idaho, Oregon, and Washington.

**Source:** National Center for Health Statistics, National Vital Statistics System, Mortality Data, 2021. https://www.cdc.gov/nchs/nvss/deaths.htm

